# Mammalian SWI/SNF Chromatin Remodeling Complexes in Embryonic Stem Cells: Regulating the Balance Between Pluripotency and Differentiation

**DOI:** 10.3389/fcell.2020.626383

**Published:** 2021-01-18

**Authors:** Ying Ye, Xi Chen, Wensheng Zhang

**Affiliations:** ^1^Cam-Su Genomic Resource Center, Medical College of Soochow University, Suzhou, China; ^2^Department of Biology, Southern University of Science and Technology, Shenzhen, China

**Keywords:** SWI/SNF (BAF) complex, embryonic stem cells, pluripotency, differentiation, chromatin remodeling complex

## Abstract

The unique capability of embryonic stem cells (ESCs) to maintain and adjust the equilibrium between self-renewal and multi-lineage cellular differentiation contributes indispensably to the integrity of all developmental processes, leading to the advent of an organism in its adult form. The ESC fate decision to favor self-renewal or differentiation into specific cellular lineages largely depends on transcriptome modulations through gene expression regulations. Chromatin remodeling complexes play instrumental roles to promote chromatin structural changes resulting in gene expression changes that are key to the ESC fate choices governing the equilibrium between pluripotency and differentiation. BAF (Brg/Brahma-associated factors) or mammalian SWI/SNF complexes employ energy generated by ATP hydrolysis to change chromatin states, thereby governing the accessibility of transcriptional regulators that ultimately affect transcriptome and cell fate. Interestingly, the requirement of BAF complex in self-renewal and differentiation of ESCs has been recently shown by genetic studies through gene expression modulations of various BAF components in ESCs, although the precise molecular mechanisms by which BAF complex influences ESC fate choice remain largely underexplored. This review surveys these recent progresses of BAF complex on ESC functions, with a focus on its role of conditioning the pluripotency and differentiation balance of ESCs. A discussion of the mechanistic bases underlying the genetic requirements for BAF in ESC biology as well as the outcomes of its interplays with key transcription factors or other chromatin remodelers in ESCs will be highlighted.

## Fundamentals of Embryonic Stem Cells

Embryonic stem (ES) cells are pluripotent cells derived from the inner cell mass of blastocyst-stage embryos (Evans and Kaufman, 1981; Martin, 1981; Thomson et al., 1998). Their importance to basic biology and translational medicine derives from two unique characteristics that distinguish them from all other cell types. First, they can be maintained as a self-renewing stem cell population *in vitro*. Second, they have the capacity to differentiate into every cell type of the body. For decades, the mechanism underlying the self-renewal and pluripotency of ESCs has been the focus of intensive research in the field of stem cell biology.

Mouse ESCs were initially established and maintained by co-culture with mouse embryonic fibroblasts (Evans and Kaufman, 1981; Martin, 1981). Subsequent studies identified leukemia inhibitory factor (LIF) as one of the feeder-cell-derived molecules that support the growth of undifferentiated ESCs through gp130-mediated activation of STAT3 (Smith et al., 1988; Williams et al., 1988; Stewart et al., 1992; Niwa et al., 1998; Matsuda et al., 1999). In contrast to mouse ESCs, LIF and STAT3 appear to be dispensable for the self-renewal of primed human ESCs (Thomson et al., 1998; Reubinoff et al., 2000; Dahéron et al., 2004). Furthermore, serum could be replaced by BMP4, which activates Smad and subsequently induces the expressions of helix–loop–helix ID factors (Ying et al., 2003). ESCs cultivated in a serum-free medium with MAPK/ERK pathway inhibitor PD0325901 and glycogen synthase kinase 3β (GSK3β) pathway inhibitor CHIR99021 (called 2i), and LIF represent naïve state and exhibit greater and homogenous pluripotent gene expression than those cultivated in serum with LIF (Ying et al., 2008). With these developments, it is now possible to grow ESCs with defined factors in the absence of serum or feeder cells.

Numerous studies demonstrate the importance of transcription factors (TFs) on the maintenance of ESCs and their pluripotency, among which OCT4, SOX2, and NANOG form a core transcriptional regulatory circuit (Martello and Smith, 2014). Ablation of their expression disrupts the pluripotency network, leading to the exit from pluripotency and initiation of differentiation of ESCs (Okamoto et al., 1990; Schöler et al., 1990; Nichols et al., 1998; Chambers et al., 2003; Mitsui et al., 2003; Masui et al., 2007; Silva et al., 2009). In addition, downregulation of epiblast-specific TFs such as TBX3, KLF2/4/5, TFCP2L1, and ESRRB disturb the self-renewal of ESCs, demonstrating their supporting roles in the maintenance of ESC identity (Ivanova et al., 2006; Jiang et al., 2008; Festuccia et al., 2012, 2018; Martello et al., 2013; Yeo et al., 2014).

## Chromatin Remodeling Complex

Besides signaling and TFs, chromatin remodeling complexes play instrumental roles on maintaining the identity of ESCs (Papatsenko et al., 2018). At least three epigenetic mechanisms allow regulation of DNA expression and chromatin accessibility, which include DNA methylation (Winata et al., 2018), histone modifications (Lawrence et al., 2016), and ATP-dependent chromatin remodeling (Clapier et al., 2017). This mini-review will focus on the SWI/SNF family of ATP-dependent chromatin remodeling complexes and its role in the maintenance of ESCs and their differentiation.

The ATP-dependent SWI/SNF complexes were first discovered in yeast in genetic screens aimed at uncovering factors responsible for the regulation of mating type switching (Stern et al., 1984) and those being able to allow changing of nutrient sources used for energy supply (Carlson et al., 1981; Neigeborn and Carlson, 1984, 1987), therefore termed SWI/SNF complex (short for SWItch/sucrose non-fermentable) (Alfert et al., 2019). In *Drosophila melanogaster*, this complex was first discovered in screens to uncover genes that are able to suppress phenotypes caused by mutations in Polycomb genes (PcGs) (Tamkun et al., 1992; Elfring et al., 1994).

The BAF (BRG1/BRM-associated factor) complex, the mammalian homolog of the SWI/SNF complex, is one of four ATP-dependent chromatin remodeling complex families known in mammals (the other three are INO80/SWR1, ISWI, and CHD complexes) (Clapier and Cairns, 2009). Three mammalian BAF complexes have been identified based on their different subunit compositions. The subunits are encoded by 29 genes (Centore et al., 2020). The PBAF (Polybromo-associated BAF complex) is distinguished from the cBAF (canonical BAF complex) by the incorporation of BAF200 instead of BAF250A/B and of BAF180 (Yan et al., 2005). Furthermore, PBAF lacks SS18 but includes the PBAF-specific subunits BAF45A and BRD7 (Kaeser et al., 2008; Middeljans et al., 2012). Recently, a third class, called ncBAF (for non-canonical BAF complex) or GBAF (after its distinctive subunits GLTSCR1/1L), has been identified, which is characterized by the incorporation of BRD9 and GLTSCR1/1L (Alpsoy and Dykhuizen, 2018), but lacks the cBAF subunits such as BAF47, BAF57, and BAF250 and the PBAF-specific subunits BAF180 and BRD7 (Clapier et al., 2017; Alpsoy and Dykhuizen, 2018; Mashtalir et al., 2018).

## Function of BAF Components in Embryonic Stem Cells

BAF complexes are made up of multiple subunits that are assembled in a combinatorial manner to tailor their functions, regulating specific developmental events (Ho and Crabtree, 2010). The BAF complexes in different tissues are distinctive for their specific subunit compositions (Lickert et al., 2004; Lessard et al., 2007; Vogel-Ciernia et al., 2013; Harada et al., 2017; Sokpor et al., 2018; Akerberg and Pu, 2020). Hence, it is not only the BAF complex itself that controls biological processes, but the expressions of distinct BAF complexes with unique subunit compositions are also a major part of the regulatory process.

The assembly of an ESC-specific BAF (esBAF) complex is required for the regulation of the ESC transcriptome, therefore controlling the self-renewal and differentiation of ESCs (Ho et al., 2009a). The esBAF complex depends on BRG1 as the ATPase subunit, as BRM does not express in ESCs (Ho et al., 2009b). Moreover, esBAF can be distinguished by the incorporation of Baf250a not 250b, Baf60a/b not 60c, and a Baf155::155 homodimer instead of a Baf155::170 heterodimer (Kaeser et al., 2008; Ho et al., 2009b). In human ESCs, BAF170, and not BAF155, seems to play an important role in the maintenance of pluripotency (Zhang et al., 2014).

A possible way of elucidating the role and importance of individual subunits of multiprotein complexes is the deletion or downregulation of genes encoding their subunits. Genetic inactivation of specific subunit of BAF complex leads to diverse aberrant phenotypes in ESCs (Table 1).

**Table 1:** BAF subunits and their role in embryonic stem cells (ESCs).

**Subunit**	**Type of mutant**	**Phenotype**	**References**
BAF250a (SMARCF1)	*Baf250a^*fl*/−^* mES cells	Inhibit self-renewal, promote differentiation into primitive endoderm-like cells, are defective in differentiating into fully functional mesoderm-derived cardiomyocytes and adipocytes, but are capable of differentiating into ectoderm-derived neurons.	Gao et al., 2008
BAF250b	*Baf250b^−/−^* mES cells	Reduced proliferation rate and an abnormal cell cycle. Deficient in the self-renewal capacity of undifferentiated ES cells and exhibit certain phenotypes of differentiated cells.	Yan et al., 2008
BRG1 (SMARCA4)	*Brg1* shRNA; *Brg1^*fl*/*fl*^* mES cells	Essential for ES cell self-renewal and pluripotency genes, and upregulation of differentiation genes.	Ho et al., 2009b; Kidder et al., 2009
DPF2 (BAF45d)	*Dpf2^*fl*/*fl*^* mES cells	Impaired meso-endoderm differentiation but promoted neuro-ectoderm differentiation.	Zhang et al., 2019
Srg3(BAF155) (SMARCC1)	*Srg3^−/−^*; *Baf155* shRNA mES cells	Mutant blastocysts hatch, adhere, and form a layer of trophoblast giant cells, degenerated inner cell mass after prolonged culture, facilitate ESC differentiation; decrease proliferation; and increase apoptosis of ES cells.	Kim et al., 2001; Ho et al., 2009b; Schaniel et al., 2009
BAF47 (SNF5) (SMARCB1)	*SNF5/INI1* null mouse embryos; *Baf47* shRNA and ectopic expression	Die between 3.5 and 5.5 days postcoitum; and Ini1-null blastocysts fail to hatch, form the trophectoderm, or expand the inner cell mass when cultured *in vitro*; knockdown Baf47 block differentiation; overexpression of Baf47 enhances differentiation of mES cells.	Guidi et al., 2001; You et al., 2013; Sakakura et al., 2019
BAF53a	*Baf53a* knockdown; *Baf53a* cKO mES cells	Cell growth repressed, induced cell death and reduction of mouse ES cell viability; Baf53b rescued cell death of Baf53a-deficient mES cells.	Zhu et al., 2017
BRD9	*Brd9* shRNA, BRD9 inhibitor	Preserving the naïve pluripotency of mouse ESCs and preventing transition to the primed state.	Gatchalian et al., 2018
BAF170 (SMARCC2)	BAF170 ectopic expression	Defects in pluripotency of mouse ES cells.	Ho et al., 2009b
BAF60c (SMARCD3)	*Baf60c* knockdown	Impaired anterior/secondary heart field, and abnormal cardiac and skeletal muscle differentiation.	Lickert et al., 2004
hBAF250a	*hBaf250a^−/−^*	Disrupted cardiomyocyte differentiation.	Lei et al., 2020
hBRG1, hBAF170	*hBrg1, Baf170* knockdown human ESCs	Defects in self-renewal of human ES cells.	Zhang et al., 2014

Both *Brg1* and *Baf155* knockout mice are lethal at the pre-implantation stage (Bultman et al., 2000; Kim et al., 2001), suggesting that they play a key role in the maintenance of pluripotency. Consistently, depletion of either *Brg1* or *Baf155* expression in ESCs leads to the downregulation of the key pluripotent TFs *Oct4, Sox2*, and *Nanog*, indicating that BAF155 and BRG1 cooperate to maintain ESC identity (Fazzio et al., 2008; Ho et al., 2009b; Kidder et al., 2009). Corresponding to the unique subunit composition of esBAF, neither *Brm* nor *Baf170* overexpression can rescue *Brg1* or *Baf155* knockout, respectively (Ho et al., 2009b). Different from mouse esBAF complex, human *Baf170* deficiency led to the differentiation of human ESCs, demonstrating that the BAF170-containing BAF complex was required for the self-renewal of human ESCs (Zhang et al., 2014). Ho et al. reported that neuro-ectodermal differentiation was impaired and mesodermal differentiation was delayed in *Brg1* knockout embryoid bodies (Ho et al., 2009a). In contrast, knockdown of *Brg1* in ESCs promoted the expression of differentiation marker genes (Kim et al., 2001). These results might indicate the distinct role of BRG1 in ESCs and differentiating cells. *Baf47* knockout mice are also lethal at the pre-implantation stage (Klochendler-Yeivin et al., 2000; Guidi et al., 2001). The negative regulation of *Oct4* by *Baf47* may control the balance between pluripotency and differentiation of ESCs (You et al., 2013). A recent contradicting report indicates the upregulation of *Cdx2* expression in *Baf47* KO ESCs (Sakakura et al., 2019). BAF250a and BAF250b are two mutually exclusive esBAF subunits. Inactivation of either of them decreases expression of *Oct4* and *Sox2* or *Nanog*, thereby inhibiting the self-renewal of ESCs (Gao et al., 2008; Yan et al., 2008). Knockout of *Baf250a* upregulates primitive endoderm maker genes, such as *Gata4, Gata6*, and *Sox17* in mouse ESCs but impairs mesodermal lineage differentiation of both mouse and human ESCs (Gao et al., 2008; Lei et al., 2020). In contrast, knockout of *Baf250b* increased the expression of mesoderm marker genes in mouse ESCs, *Gata2* and *Esx1* (Yan et al., 2008). This may indicate the balance role of BAF250a- and BAF250b-containing BAF complexes on mesoderm differentiation of ESCs. The deletion of esBAF subunit *Baf45d* only perturbs the self-renewal of ESCs, whereas its knockout impairs the differentiation of ESCs to all three germ lineages (Zhang et al., 2019).

In addition to the long-known esBAF, the newly discovered ncBAF complex also plays an important role in the regulation of the ESC transcriptome. Inhibition of *Brd9*, the specific ncBAF subunit, changed the morphology of ESCs to that resembles primed or epiblast ESCs (EpiESCs), reduced colony-forming capability, and downregulated expressions of *Nanog* and *Klf4*, indicating that BRD9 has an important role in maintaining the naïve pluripotent state of ESCs (Gatchalian et al., 2018).

Consistent to its functions on the maintenance and differentiation of ESCs, BAF complexes also play important roles in the reprogramming of somatic cells to induced pluripotent stem cells (iPSCs). Depletion of *Brg1* was associated with failures in reprogramming (Hansis et al., 2004; Egli and Eggan, 2010). Overexpression of *Brg1* and *Baf155* achieves euchromatin, enhances binding of OCT4, and increases the reprogramming efficiency of MEFs to iPSCs (Singhal et al., 2010). In contrast, downregulation of *Brm* and *Baf170* improves reprogramming efficiency and promotes complete reprogramming of immature iPSCs (Jiang et al., 2015). Therefore, similar to the distinct roles of different BAF subunits for the maintenance and differentiation of ESCs, different BAF components may play different roles in the reprogramming.

In summary, esBAF complex is crucial for the maintenance of ESCs, with distinct effects from the deletion of different subunits. The knockout of different subunits of esBAF leads to defects of ES differentiations to different lineages, though the precise molecular mechanisms underlying the different phenotypes upon the deletion of different subunits need further investigations.

## Mechanistic Insights Into BAF Complexes in Embryonic Stem Cells

Inactivation of individual esBAF subunits downregulates expression of pluripotent TFs (Gao et al., 2008; Yan et al., 2008; Ho et al., 2009b; You et al., 2013; Zhang et al., 2019), indicating that esBAF controls the self-renewal of ESCs via regulating pluripotent factors. esBAF subunits BRG1, BAF155, BAF250a, and BAF45d are bound at sites engaged by the core pluripotency TFs OCT4, SOX2, and NANOG (Ho et al., 2009a; Gatchalian et al., 2018; Zhang et al., 2019). The expression of the core TFs *Nanog, Oct4*, and *Sox2* as well as a variety of other factors governs the maintenance of pluripotency in ESCs (Martello and Smith, 2014). Specifically, NANOG, OCT4, and SOX2 have been shown to repress the expression of developmental genes while modulating their own expression levels by binding to each other's promoter regions (Saunders et al., 2013). Both BRG1 and BAF155 are located near the transcriptional starting site (TSS) of core pluripotency factors *Oct4, Nanog*, and *Sox2* (Ho et al., 2009b). The binding of OCT4, SOX2, and NANOG is impaired in *Dpf2* KO ESCs (Zhang et al., 2019). Therefore, esBAF complex may collaborate with the core TFs to regulate the expression of pluripotency TFs, thereby controlling the maintenance of ES self-renewal.

LIF/STAT3 pathway is essential for the maintenance of mouse ESCs (Niwa et al., 1998; Matsuda et al., 1999). It also plays a role in naïve or murine-like human ESC pluripotency (Hanna et al., 2010; Buecker et al., 2014). BRG1, DPF2, and STAT3 binding sites display a substantial genome-wide overlap in mouse ESCs. STAT3 binding is considerably impaired in *Brg1*- or *Dpf2* (*Baf45d*)-depleted ESCs, leading to the downregulation of *Stat3* target genes (Ho et al., 2011; Zhang et al., 2019). esBAF stabilizes the binding of STAT3 and thereby helps the maintenance of ES self-renewal (Ho et al., 2011).

esBAF also regulates gene expression in ESCs (Ho et al., 2011; Gatchalian et al., 2018; Zhang et al., 2019). esBAF preferably binds to enhancers and regulates their H3K27ac deposition. Loss of esBAF subunit Dpf2 changes the activity of enhancers and the target gene expression (Ho et al., 2011; Zhang et al., 2019). On the other hand, ncBAF predominantly binds to H3K4-trimethylated promoter regions and is associated with the TFs *Klf4* (Kruppel-like factor 4) (Gatchalian et al., 2018), indicating a distinct mechanism to regulate gene expression. One of the most striking differences is, however, that ncBAF binds to TAD (topologically associating domain) boundaries and CTCF sites, potentially contributing to the regulation of genome topology (Gatchalian et al., 2018). Thus, esBAF and ncBAF complexes might regulate ESC identity coordinately via distinct mechanisms that future studies need to elucidate.

## Collaboration of BAF Complex With PRC2 Complex in Embryonic Stem Cells

The PcG family has first been discovered in *Drosophila* followed by the observation of male flies with ectopic sex combs (Margueron and Reinberg, 2011). In mammals, the multiprotein-containing Polycomb repressive complex 2 (PRC2) has repressive influence on the genome (Margueron and Reinberg, 2011). PRC2 is dispensable for the maintenance of undifferentiated mouse ESCs, as the deletion of PRC2 components has little effect on their morphology and self-renewal, although a subset of PRC2 target genes are derepressed (Boyer et al., 2006; Pasini et al., 2007; Chamberlain et al., 2008; Shen et al., 2008; Leeb et al., 2010). Similarly, deletion of EZH2, the catalytic subunit of PRC2 complex, in human ESCs also causes misexpression of developmental genes but severely affects the self-renewal of human ESCs (Collinson et al., 2016).

The cooperative function of BAF complex with PRC2 in ESCs has been revealed (Ho et al., 2011; Zhang et al., 2019). Ho et al. report that the core subunit of BAF complex, BRG1, in ESCs potentiates LIF signaling by opposing PRC2 complex (Ho et al., 2011). The opposing regulation of BAF and PRC2 subunits DPF2 and EED on *Tbx3* expression is critical to the proper differentiation of ESCs to mesoendoderm. The other PRC2 subunit EZH2 also opposes DPF2-dependent differentiation through a distinct mechanism involving *Nanog* repression (Zhang et al., 2019). Contrary to the opposing function of BAF and PRC2 complexes, BRG1 facilitates PRC2 to reinforce the repression on its target genes in ESCs. Therefore, esBAF not only simply antagonizes PcG but also acts synergistically with the common goal of supporting pluripotency (Ho et al., 2011).

**Figure 1:**
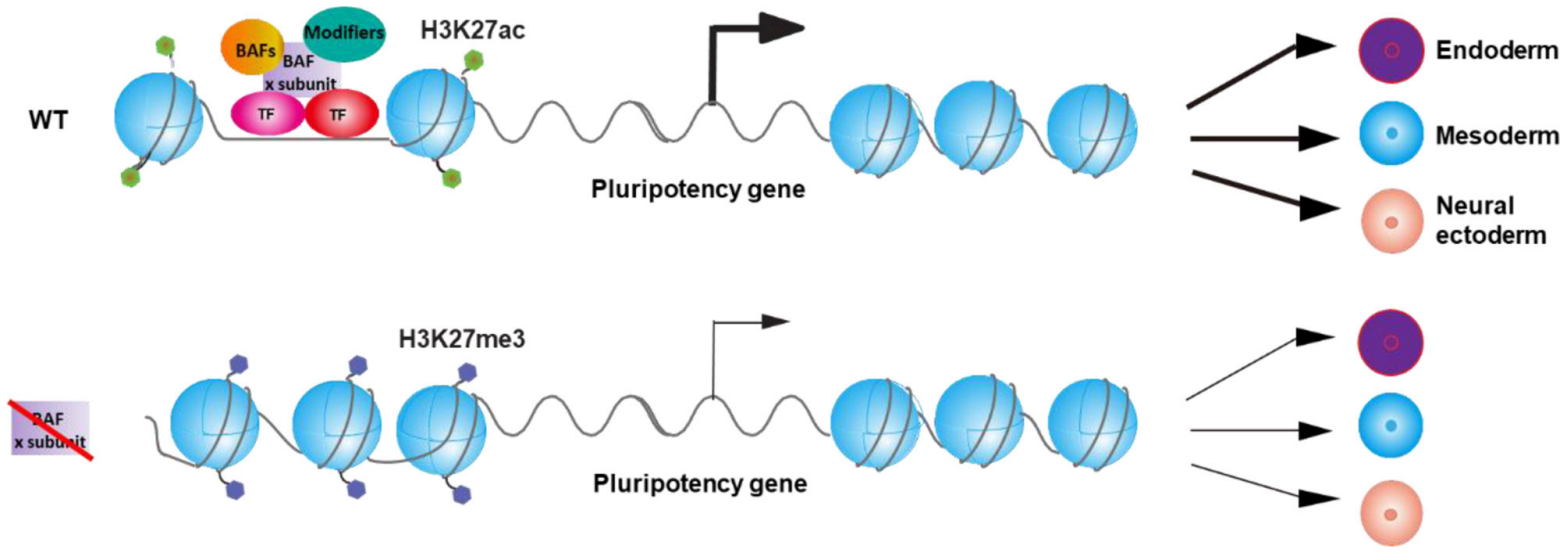
Model for the regulation of the balance between pluripotency and differentiation of embryonic stem cells (ESCs) by BAF complexes *via* the control of pluripotency gene expression. BAF complex, transcription factors, and other chromatin modifiers regulate the expression of specific pluripotency gene(s) and thereby control the balance between pluripotency and differentiation of ESCs. Inactivation of specific BAF subunit leads to the deregulation of the expression of specific pluripotency gene(s) and therefore results in the differential differentiation defects of ESC.

Inactivation of different subunit of BAF complex differentially affects the expression of pluripotency TFs (Table 1). Furthermore, DPF2 opposingly regulates differentiation of ESCs via controlling different pluripotency TFs with distinct components of PRC2 complex. As a result, distinct BAF subunits may regulate the expression of different pluripotency TFs collaboratively with other TFs and chromatin modifiers and therefore leads to different differentiation defects of ESCs upon the deletion of different BAF components (Figure 1). Interestingly, knockout of *Dpf2* only affects about 8% of BRG1 binding sites on the genome (Zhang et al., 2019), indicating that the loss of a specific BAF subunit only affects the binding of a specific portion of BAF complex on genome, which may lead to the specific phenotypes upon the deletion of that subunit. Future studies on the deletion of other specific BAF components on the binding of BRG1 will help to explain the different phenotypes in ESCs that resulted from the deletion of specific subunits of other chromatin complexes.

### BAF Complex on the Balance Between Pluripotency and Differentiation

BAF complex regulates both the maintenance and differentiation of ESCs (Ho and Crabtree, 2010). Knockout of *Dpf2* does not change the level of H3K27ac around DPF2-bound lineage markers during differentiation of ESCs. Consistently, overexpression of Dpf2 in ESCs does not lead to the upregulation of endo- and mesodermal markers, supporting an idea that BAF complex regulates ESC differentiation indirectly (Zhang et al., 2019). *Tbx3* is a pluripotency TF, and the downregulation of its expression impairs ESC self-renewal (Ivanova et al., 2006). *Tbx3* also plays key roles on ESC differentiation. Deregulation of its expression impairs the differentiation of ESCs (Lu et al., 2011; Weidgang et al., 2014; Waghray et al., 2015; Zhang et al., 2019). *Dpf2* participates in the self-renewal and differentiation of ESCs via precisely regulating *Tbx3* expression in both ESCs and differentiating cells (Zhang et al., 2019). As a core pluripotency TF, *Nanog* represses expression of differentiation marker genes and maintains the self-renewal of ESCs (Niwa, 2007). *Dpf2* regulates the expression of *Nanog* with PRC2 subunit *Ezh2*, thereby controlling the proper differentiation of ESCs (Zhang et al., 2019).

BAF47 controls the differentiation of ESCs via regulating *Oct4* expression, which provides another example to demonstrate how BAF complex controls the balance between pluripotency and differentiation (You et al., 2013). The controversial result from a recent work upon the deletion of *Baf47* in ESCs indicates that more studies are required to clarify the discrepancy (Sakakura et al., 2019). Changed expression of pluripotency genes in ESCs upon the deletion of other BAF subunits has been reported (Gao et al., 2008; Ho et al., 2009b; Ho and Crabtree, 2010). It will be of interest to carry out systematic studies to determine whether and how other subunits of BAF complex regulate the expression of specific pluripotency genes and thereby control the balance between pluripotency and differentiation.

## Conclusion

BAF complex is functionally important for the self-renewal and differentiation of ESCs. Knockout of different subunits of BAF complex changes the expression of different pluripotency TFs and impairs the differentiation of ESCs differently. Thus, it is of particular importance to explore how BAF complex regulates the balance between the maintenance of identity of ESCs and their differentiation to three germ layers. We have outlined studies that described functions of specific subunits of various BAF complexes in ESCs. Moreover, our recent study demonstrates an attractive mechanism that distinct BAF subunit controls the integrity of only a part of BAF complex on the genome, and therefore, its deletion only affects the binding of a part of BAF complex, which directly changes the expression of distinct pluripotency TFs in both ESCs and differentiating cells with other TFs and chromatin modifiers (Zhang et al., 2019). Consistently, another recent report demonstrates that the loss of a single subunit of the BAF complex in cancer cells did not destroy the entire complex but will change the composition of the BAF complex (Schick et al., 2019). BAF complex regulates ESC differentiation via controlling the expressions of pluripotency TFs, with different subunits affecting ESC differentiation via regulating different TFs. Further systematic studies of other subunits of BAF complex are needed to warrant the mechanism, which may also explain the distinct phenotypes that resulted from the deletion of various subunits of chromatin remodeling complex.

## Author Contributions

YY, XC, and WZ conceived the study and wrote the manuscript. All authors contributed to the article and approved the submitted version.

## Conflict of Interest

The authors declare that the research was conducted in the absence of any commercial or financial relationships that could be construed as a potential conflict of interest.
